# Supplementary material for: Socioeconomic effects of water hyacinth (Echhornia Crassipes) in Lake Tana, North Western Ethiopia
Source: PLoS One. 2020 Sep 2;15(9):e0237668. doi: 10.1371/journal.pone.0237668 (PMC7467254; doi:10.1371/journal.pone.0237668)
Supplement: S1 Questionnaire — (PDF) [file pone.0237668.s001.pdf]

Questionnaire for socio-economic impact of water hyacinth

My name is ……………………………………………….……. I am from Bahir Dar University. We have planned to conduct a study on the Socio-Economic Impact of Water Hyacinth around Lake Tana. The objective of this study is to evaluate the social and economic impacts of water hyacinth in some selected woredas of Lake Tana Area . We have chosen randomly a list of households from your village. We were able to find your name through the records of development agents/kebele administration. We will ask some questions on your land management practices, agricultural production, livelihood activities and others. We kindly requested you to answer every question with the facts to the best of your knowledge. Your kind cooperation has vital for the success of this study and will be therefore highly appreciated. Your responses will be restricted to our study use only. Your name will not appear in any public documents. Thank you very much for your cooperation.

1. **Identification**

No		Code
1.1.	Name of *Woreda*	
1.2.	Name of kebele administration	
1.3.	Name of Village	
1.4.	Name of the head of household	
1.5.	Date of survey	
1.6.	Name of enumerator	
1.7.	Field Supervisor	

2. **Demographic and Social Characteristics**

Age of respondent (in years)	Sex of respondent:<br>1. Male<br>2. Female	Educational level<br>1. None<br>2. 1-4<br>3. 5-8<br>4. 9-10<br>5. 11-12<br>6. TVET<br>7. University	Number of members in the household<br>Male _______<br>Female_____<br>Total____	Marital status<br>1. Married or living together<br>2. Single<br>3. Divorced/separated<br>4. Widowed/widow

3. **Ownership and use of Lands**

3.1. Does your household own any land? Yes [1] No [2]

3.2. How many *timads*/*gemed* /*kadas* of land do your household own now? __________________

3.3. If yes, in which location does your household own? Homestead [1] dry farmstead [2] lakeshore [3] Within wetland [4] others

3.4. How many plots of land do you own? Please specify their location, area, uses, etc, in the table below

Location of plot	Number of plots	Total area (in *timad/kada*)	What are the plots used for?<br>1. Arable land<br>2. Grazing land	How did you acquire the land you own?<br>1. Inherited<br>2. Rented -in<br>3. Squatter<br>4. land redistribution<br>5. Share cropped-in	How many years have you cultivated the plot?
1. Homestead					
2. Dry farmstead					
3. Lakeshore					
4. Wetland					
5. Others					

4. Livestock Ownership

4.1. Does your household own livestock? Yes [1] No [2]

4.2. If yes, please mention the types and number of livestock you own and the trends of ownership the following table

S. No	Livestock	Total Number of owned	The trend of the number in the past 10 years<br>Increased 1<br>Decreased 2<br>No change 3
1.	Oxen		
2.	Cows		
3.	Bulls		
4.	Heifers		
5.	Calves		
6.	Sheep		
7.	Goats		
8.	Horses		
9.	Donkeys		
10.	Mules		
11.	Chickens		
12.	Beehives		

5. Source of Animal Feed/Grazing

5.1. What are the main sources of animal feed? Grazing [1] Fodder trees and grasses [2] Hay [3] by products [4] others [5]

5.2. If the major sources of your animal feed is grazing, please indicate its location, status and trends in the table below

Location of animal feed sources	Type of feed/grazing [use key 1)	The current status<br>Abundant 1<br>Limited 2	The trends in the past five years<br>Increased 1<br>Decreased 2<br>No change 3
1. homestead			
2. dry communal land			
3. wetland and lakeshore			
4. dry land farmsteads			
5. others			

6. Livelihood Activities and Income Sources

6.1. Does your household involve in off-and non-farm activities other than agricultural production? Yes [1] No [2]

6.2. If yes, please give the details of the types of livelihood activities that your household currently engaged and state the amount you received from every livelihood activity during the last cropping season

S. No	Activity/ income source	Is your household involved?<br>1=Yes<br>2=No	income earned in the last 12 months
1.	Formal/salaried employment		
2.	Public works (safety net)		
3.	Wage labour		
4.	Trading in crops		
5.	Trading in livestock		
6.	Trading in manufactured goods		
7.	Sale of charcoal or firewood		
8.	Local alcoholic drink preparation		
9.	Basket making		
10.	Embroidery		
11.	Carpentry/house building		
12.	Pottery		
13.	Blacksmithing		
14.	Renting out land		
15.	Remittances		
16.	Fishing		
17.	Others (specify, if any)		

7. Infestation of Water hyacinth

7.1. Rate of Infestation

7.1.1. Do you know the plant called water hyacinth (*emboch*)? 1= Yes 2=No

7.1.2. If your response is yes, please indicate the infestation rate of the weeds and other issues in the following table

When did you notice this weed for the first time?<br>1=just this year<br>2=three years ago<br>3=five years ago<br>4=ten years ago	In which area does the weed grow?<br>1=Rangelands<br>2=around the village<br>3=in crop lands<br>4=within the lakes<br>5=Marshlands	Do you know how it came to your area?<br>1=by livestock<br>2=by wild animals<br>3=it just grew itself<br>4=brought by foreigners (visitors)<br>5=don't know<br>6=Others	To what extent the weed invade the grazing lands?<br>1=Very dense<br>2=medium<br>3=sparse
☐	☐	☐	☐
To what extent the weed invade crop lands?<br>1=Very dense<br>2=medium<br>3=sparse	To what extent the weed invade the lake and marshlands?<br>1=Very dense<br>2=medium<br>3=sparse		
☐	☐		

7.1.3. Has your cultivated plot/s invaded by water hyacinth? 1=Yes 2=No

7.1.4. if yes, please give the response for the following questions

How many hectares of your farmland have been invaded by this weed?	In what extent those rangelands invaded?<br>1=very dense<br>3=Medium<br>4=Sparse	Has this plant affected you agricultural activities?<br>1=Yes<br>2=No	If yes, which activities have been affected?<br>1=ploughing<br>2=weeding<br>3=harvesting<br>4=threshing	If the weed constrains these activities, to what extent has it affected?<br>1= High<br>2=Medium<br>3=Low	Has the infestation of water hyacinth increased the cost of these activities?<br>1=yes<br>2=No
☐	☐	☐	☐	☐	☐

7.2. Effect of Water Hyacinth on crops production

7.2.1. If the infestation of this weed increase the cost/labour of agricultural activities, please estimate the cost for each agricultural activities

S. No	Agricultural activities	Before infestation		After infestation	
		Labor	Cost	labor	Cost
1.	Ploughing				
2.	Weeding				
3.	Harvesting				
4.	Threshing				

5.	Others				

7.2.2. Is there any change in the productivity of your crops by the presence of water hyacinth in your farm plots?

Yes [1] No [2]

7.2.3. If yes, please estimate the yield of crops you produced before and after the infestation of water hyacinth?

S. No	Types of crops	Before infestation		After infestation		Farm gate price
		Area	Production	Area	Production	
7.3.	Teff					
7.4.	Rice					
7.5.	Maize					
7.6.	Barely					
7.7.	Wheat					
7.8.	Sorghum					
7.	Finger Millet					
8.	Faba Bean					
9.	Cow Peas					
10.	Chickpea					
11.	Vetch					
12.	Oil Seeds					
13.	Potato					
14.	Onion					
15.	Garlic					
16.	Cabbage					
17.	Tomato					
18.	Spices					
19.	Others					

7.3.Effects of water hyacinth on grazing lands

7.3.1. Has the infestation of water hyacinth on grazing lands affect your cattle feed?

Yes [1] No [2]

7.3.2. If yes, please respond the following questions

To what extent the infestation weed affect animal feeding?<br>1=Minimum<br>2=Moderate<br>3=High<br>4=Very high	How does the infestation weed affect the animal feed?<br>1=decrease the palatable grasses<br>2=prevent livestock grazing/browsing<br>3=Decrease harvesting of hay<br>4= Reduction of crop residues<br>5=Others	If your response is decreased palatable grasses, how many number of grass species lost due to the appearance of the weed?	Did you harvest hay from the field currently invaded by the weed before its appearance?<br>1=yes<br>2=no	If you harvested, could you estimate the quantity of hay harvested?

Does the infestation of the weed result for the shortage of your livestock feed?<br>1=Yes<br>2=No	If yes, please estimate the number of months that your animals faced feed shortage due to infestation of water hyacinth	What are your strategies to fill the gap created by this weed appearance?<br>1=buying of hay<br>2=buying of crop residues<br>3=buying of industrail byproducts<br>4=Leaving crop field for animal grazing<br>5=plantation fooder trees/grasses/crops<br>6=others	If you have been bought hay to adress the problem, could you estimate the cost per year	If you have been bought crop residue to adress the problem, could you estimate the cost per year
If you have been bought byproducts to adress the problem, could you estimate the cost per year	If you have left crop field for grazing to adress the problem, could you estimate the area of land?	What crops did you frequetly grow the crop field left for cattle grazing?<br>1=Rice<br>2=Teff<br>3=Chickpea<br>4=Vetch<br>5=Spices<br>6=Vegetables<br>7=others	Could you estimate the quantity of crop residue reduced because of the appearance of this weed?	

7.4.Effects of WH on fishing activities

7.4.1. Does the water hyacinth affect fishing activities in Lake Tana?

1. Yes ☐ 2. No ☐

7.4.2. If your household engaged in fishing and the catching has constrained by the weed, please respond the following questions

To what extent the infestations of the weed affect fish catching?<br>1=low<br>2=moderate<br>3=sever<br>4=very sever	What are the impacts of the weed in fishing?<br>1=decline the quality of fish<br>2=construing boat transport<br>3=decrease the fish stock by blocking access breeding &feed<br>4=Obstruct netting<br>5=decrease the life span of nets<br>6=slowing fish growth<br>7=Others	If fish stock decrease, why?<br>1=the feed of fish is declined by the weed<br>2=the weed kills fish<br>3=the weed constrained the fish migration to breeding habitat<br>4=others	How many fishing gears did you buy a year when water hyacinth was absent	How many gears do you buy per year after the infestation of the weed?

How much does fishing gear cost?	Have you noticed the difference in terms of the quantity of fish catched before &after infestation<br>1=Yes<br>2=No	How many kilograms of fish did you make before infestation?	How much was it sold then?	How many kilograms do you catch now?
How much do you sell a kilogram of fish currently?	Have you observed the difference in terms of fish species before & after the infestation of the weed?<br>1=yes<br>2=no	If yes, which species of fish did you usually catch before the weed appeared?<br>1=White fish<br>2=Koroso<br>3=Ambaza<br>4=Bezo	Which species have you usually catched since the weed appeared?<br>1=White fish<br>2=Koroso<br>3=Ambaza<br>4=Bezo	Which kind of fish species disappeared or diminished with the appearance of water hyacinth?<br>1=White fish<br>2=Koroso<br>3=Ambaza<br>4=Bezo

7.5.Effects of water hyacinth on sand extraction

7.5.1. If your household engaged in sand extraction, is there any impact on the extraction of sand? Yes 2. No

7.5.2. If your response for Q 14 is "yes", please respond the following questions

How far the infestation of water hyacinth affects your sand extraction?<br>1=Nothing<br>2=Low<br>3=Medium<br>4=High	How these weed infestations affect the extraction of sand?<br>1=obstructing extraction<br>2=obstructing vehicles<br>3=increasing the labor<br>4=Others	Would you estimate the quantity of sand extracted before the weed appeared	How much money did you earn before the weed appeared on lakeshore?	Would you estimate the quantity of sand extracted after infestation	How much money does your household earn per year after infestation

7.6.Effects of water hyacinth on water supply

7.6.1. Please indicate the source of water for drinking and other purposes in the following table

Purpose	The source of water<br>1=Public tab<br>2=Protected dug well<br>3=Unprotected dug well<br>4=Protected spring<br>5=Unprotected spring<br>6=Rain water	Does the water hyacinth affect the supply of water?<br>1=Yes<br>2=No	If yes, what are the impacts?<br>1=the quality of water decreased<br>2=the quantity of water decreased<br>3=caused for the shortage of water<br>4= Increased the burden on	If caused for the shortage of water, how many months have you faced after?

	7=River/stream<br>8=Pond<br>9=Lake<br>10=Marshes<br>11=Others		fetching of water<br>5=Prevent the movement of cattle<br>6=Others (specify, if any)	
Drinking water				
Washing utensils				
Washing clothes				
Bathing				
Cooking				
Livestock water				
Swimming				

7.7.Effects of water hyacinth on human and animal health

7.7.1. What kinds of health problems are common in your local area?

Choler ☐ 2. Malaria ☐ 3. Typhoid ☐ 4. Diahria

5. Others (specify, if any)

7.7.2. How has the infestation of water hyacinth affected the prevalence of these diseases?

Increased ☐ 2. Decrease ☐ 3. No effect ☐ 4. No ideas ☐

7.7.3. If the prevalence of such diseases increased because of the appearance of water hyacinth, has any member of your household got sick?

Yes ☐ 2. No ☐

7.7.4. If yes, did any of the family members visit hospital/ health center/clinic?

Yes ☐ 2. No ☐

7.7.5. How much does the treatment of these diseases cost?

7.7.6. Are snakes and other ferocious wild animals common in lake and lakeshores since water hyacinth appeared? 1. Yes ☐ 2. No ☐

7.7.7. Were they common when there was no water hyacinth? 1. Yes ☐ 2. Yes ☐

7.7.8. How frequent do you have cases of attacks from snakes and others after the weed appeared?

None ☐ 2. Rarely ☐ 3. Severely ☐

7.7.9. Have you ever noticed any health problems to your cattle due to its presence?

Yes ☐ 2. No ☐

7.7.10. If yes, have your animal affected by animal diseases outbreak because of the infestation of water hyacinth? 1. Y ☐ 2. No ☐

7.7.11. If yes, could you list the diseases affected your animals

7.7.12. Did you bring your cattle affected by the diseases to veterinary clinics? 1. Yes 2. No

7.7.13. If yes, how much does the treatment of these diseases cost per head/year?

7.8.Effect of water hyacinth on wetland resources utilization

7.8.1. Have you used wetland resources for various purposes? 1. Yes 2. No

7.8.2. If your answer is 1 or others, please give response for the following questions

For what purpose has your household harvested the macrophytes	types of macrophyte 1=papyrus 2=Filla 3=ketema 4=shrubs 5=others	Was the use commercial, subsistence or both?	If commercial, how much money did you get per year before weed appearance	Does the appearance of the weed affect the quantity?	If yes, could you estimate the amount of money your household lost due to disappearance of macrophyte
Fuel					
Edible fruits					
Herbal medicines					
Making of mats					
Thatching or roofing					
Making of ropes					
Making of Basket					
Making of reeds					
Fodder for cattle					
Thatch grass					
Chefe					
Others					

8. Controlling Mechanisms

8.1.Is there any effort in controlling the expansion of water hyacinth in your local area?

1. Yes ☐ 2. No ☐

8.2.If yes, please give response for the following questions

Which mechanisms did apply to eradicate the weed from communal land? 1=Physical control 2=Mechanical control 3=Chemical control 4=Biological control 5=Utilize the plant	In what way has the community made effort to control the weed? 1=Through campaign 2=Individually 3=Payment	If campaign, who coordinate it? 1=Regional 2=Woreda government 3=KA 3=The community 4=NGOs 5=Others	Has any member of household taken part in eradicating of the weed from communal land? 1=yes 2=no	If yes, how many members of household participated in the eradication campaign?
☐	☐	☐	☐	☐
For how many days have they participated per year?	How the weed has removed from the farm holding of individual farmers 1=through campaign 2=Farmers themselves	Have you ever controlled the weed from your farm plots? 1=yes 2=no	If yes, which controlling mechanisms have you used? 1=Physical 2=Chemical 3=utilize the plant	If you used chemicals (pesticides), would you estimate the cost
☐	☐	☐	☐	☐

9. Benefits of Water Hyacinth

9.1.Does water hyacinth have any benefit in your local area? 1=yes 2=no

9.2.If yes, please give response for the following questions

What are the benefits of this weed in the local area?<br>1=animal fodder<br>2=Basket work<br>3=Making of Chair<br>4=pollution control<br>5=Compost preparation<br>6=Biogas production<br>7=Paper making<br>8=Others	Have you used this weed for any purpose?<br>1=yes<br>2=no	If you used, for what purpose?<br>1=animal fodder<br>2=Basket making<br>3=making of chair<br>4=compost preparation<br>5=Biogas production<br>6=Others	If you used it, how far it narrows your livestock feed gap?<br>1=Nothing<br>2=low<br>3=Moderately<br>4=High	Please estimate the expenditure your household saved due to the use of the weed for livestock fodder
If your household generate income by making hand crafts, could you estimate the income you earn per year	If you prepare compost, could you estimate the money you saved from purchasing of chemical fertilizers	Have you noticed the increment of yield due to the presence of this weed?<br>1=yes<br>2=no	If yes, could you estimate the yield difference after weed appearance	Have far the previous measures taken either by individually or through campaign control water hyacinth?<br>1=increased<br>2=decreased<br>3=No change
If the infestation of the weed increased, why?<br>1=Inadequate efforts<br>2=the reproductive nature of the weed<br>3=less effectiveness of controlling measures<br>4=others	What kind of controlling mechanisms should be taken?			

Thank You
