# Supplementary material for: Socioeconomic effects of water hyacinth (Echhornia Crassipes) in Lake Tana, North Western Ethiopia
Source: PLoS One. 2020 Sep 2;15(9):e0237668. doi: 10.1371/journal.pone.0237668 (PMC7467254; doi:10.1371/journal.pone.0237668)
Supplement: Attachment — Submitted filename: Response to Reviewers.docx [file pone.0237668.s003.docx]

**Response to Reviewers**

**Reviewer 1**

1. There are still several quotes of FGDs in the manuscript. Reviewer and editor suggested to delete them and include main message in running text. This will make the manuscript concise.

**Response:** Four out of five quotes of FGDs in the manuscript are deleted and included in the main message in running text. We believe that one of FGDs quotes is put as it is to provide a picture on the issue.

2. Line 46: It is good to use IAPS instead of IAS for the Invasive Alien Plant Species.

**Response**: IAS changed to IAPS

3. Line 107-109: Geographic range (latitude and longitude) given in the text and map (Figure 1) does not match. It appears that the text description is true for Lake Tana Basin Boundary, rather than Lake Tana. Please check and correct. Replace zero in superscript by symbol degree in latitude and longitude values.

**Response**: The geographical locations given in the map are written in text . Zero is replaced by symbol

4. Line 129: Scale bar in two maps seems misplaced/exchanged. Please check scale bar of each map and correct them.

**Response**: We made correction

5. Line 156: The word 'about' is probably incorrect. If 408 is the exact number of households surveyed, write this in word without 'about' here.

**Response**: Correction is made based on comments

6. Line 278: Table 1- What is HHs in second column head? Spell out as there is no space limitation.

**Response:** Done based on comments

7. Line 286-287: It is good to follow SI unit system for yield value. Please present yield values in round figure, e.g. 3052 instead of 3052.35. Do this rounding for all similar values (e.g. in line 293).

**Response**: corrections have made based on comments

8. Line 414: Spell TLU in the last column.

**Response**: TLU is spelt use Tropical Livestock Unit

9. Line 478: Table 4 - present all values in round figure without decimal.

**Response**: done

10. Line 604-605: Check figure number. You already have Fig 1 in study area section. Make title of both y axis clear. Include 'Thousands' inside parentheses.

**Response:** Corrected based on comments

11. Line 701-740: Length of conclusion can be shortened by deleting texts that repeat results.

**Response**: we tried to shortened
